# Effects of microplastic concentration, composition, and size on *Escherichia coli* biofilm-associated antimicrobial resistance

**DOI:** 10.1128/aem.02282-24

**Published:** 2025-03-11

**Authors:** Neila Gross, Johnathan Muhvich, Carly Ching, Bridget Gomez, Evan Horvath, Yanina Nahum, Muhammad H. Zaman

**Affiliations:** 1 Department of Materials Science and Engineering, Boston University 1846 , Boston, Massachusetts, USA; 2 Department of Biomedical Engineering, Boston University 168353 , Boston, Massachusetts, USA; 3 Center on Forced Displacement, Boston University 1846 , Boston, Massachusetts, USA; Centers for Disease Control and Prevention, Atlanta, Georgia, USA

**Keywords:** microplastics, antimicrobial resistance, *Escherichia coli*, public health, environmental microbiology

## Abstract

Microplastics (MPs) have emerged as a significant environmental pollutant with profound implications for public health, particularly as substrates to facilitate bacterial antimicrobial resistance (AMR). Recently, studies have shown that MPs may accommodate biofilm communities, chemical contaminants, and genetic material containing AMR genes. This study investigated the effects of MP concentration, composition, and size on the development of multidrug resistance in *Escherichia coli*. Specifically, we exposed *E. coli* to varying concentrations of different MP types, including polyethylene, polystyrene, and polypropylene, across a range of sizes (3–10, 10–50, and 500 µm). Results indicated that the biofilm cells attached to MPs had elevated multidrug resistance (in *E. coli*. Notably, MPs exhibited a higher propensity for facilitating biofilm and resistance than control substrates such as glass, likely due to their hydrophobicity, greater adsorption capacities, and surface chemistries. Notably, we found that the bacteria passaged with MPs formed stronger biofilms once the MPs were removed, which was associated with changes in motility. Thus, MPs select cells that are better at forming biofilms, which can lead to biofilm-associated AMR and recalcitrant infections in the environment and healthcare setting. Our study highlights the importance of developing effective strategies to address the challenges posed by MPs.

**IMPORTANCE:**

Antimicrobial resistance (AMR) is one of the world's most pressing global health crises. With the pipeline of antibiotics running dry, it is imperative that mitigation strategies understand the mechanisms that drive the genesis of AMR. One emerging dimension of AMR is the environment. This study highlights the relationship between a widespread environmental pollutant, microplastics (MPs), and the rise of drug-resistant bacteria. While it is known that MPs facilitate resistance through several modes (biofilm formation, plastic adsorption rates, etc.), this study fills the knowledge gap on how different types of MPs are contributing to AMR.

## INTRODUCTION

Global plastic use and mismanaged disposal are significant environmental and public health concerns. Plastic use has increased 20-fold since 1964, and prevailing estimates suggest global unmanaged trash will reach 155–265 megatons per year in 2060 (1). Not surprisingly, the detection of microplastics (MPs) has significantly increased. MPs are canonically insoluble synthetic particles or polymer matrices with regular or irregular shapes and sizes ranging from the micrometer to millimeter range (2). Primary sources of MPs include polyethylene (PE), polypropylene (PP), and polystyrene (PS) particles in cosmetic and medicinal products (3). Several countries have banned the use of primary MPs within certain industry segments; however, MPs are still generated through the degradation of existing plastics, creating contamination beyond legislation around new product formulations (3). These degradation products, characterized as secondary MPs, originate from physical, chemical, and biological processes, resulting in plastic debris fragmentation. Different sources of MPs and their varying surface chemistry also cause them to occur in varying shapes and sizes, such as pellets, fibers, and fragments in environmental samples (3). Depending on the conditions plastics are exposed to and the resulting change in surface properties, MPs arise as a unique substrate that has proved to be very difficult to control (4, 5).

MPs have infiltrated various ecosystems on the planet, ranging from submarine canyons (6) to the summit of Mount Everest (7). Additionally, wastewater has become a significant reservoir for MPs and other anthropological wastes. Despite global awareness, MPs can persist through wastewater treatment plants, disseminating them into surrounding environments. Their persistence can be attributed to their small size, buoyancy, and hydrophobic properties, allowing them to adhere to organic matter and avoid sedimentation (8). Consequently, treated wastewater effluents serve as a main source of MP pollution in aquatic environments where they accumulate in sediments and surface waters and interact with the organisms around them (8). The repercussions of MPs in wastewater are manifold, especially considering their impacts on human health.

Contemporaneously, increased rates of antimicrobial resistance (AMR)—the ability of microbes to protect themselves against antimicrobials—have been observed in bacterial populations across the globe. AMR can be influenced by a multifaceted network of factors, including the overuse and misuse of antibiotics, poor sanitation and hygiene, and environmental contamination with antibiotic residues in wastewater (9). Recent studies show that MPs might also play an important role in the development of AMR (10–12). This is mainly due to their ability to accommodate microbial communities and chemical contaminants and genetic material containing antimicrobial-resistant genes (ARGs) through biofilm formation (13). In these communities of bacteria that grow together colonizing MP surfaces, ARGs can be transferred to pathogenic bacteria through horizontal gene transfer (13). While it has been established that bacteria on the surface of MPs host ARGs, there is limited knowledge on AMR development as a function of MP properties (composition, size, concentration, etc.) as well as their interactions with various antibiotics (14, 15).

Understanding the interplay between AMR and MPs is critical, especially in places with high infection rates and significant plastic waste, such as low-resource settings. The difficulty in treating infectious diseases in these areas, combined with inadequate wastewater treatment—which may result in higher concentrations of MPs—may contribute to the observed increase in AMR cases among vulnerable populations. Therefore, understanding the fundamental interactions between MPs and AMR development is imperative. To date, few studies have looked at the effect of MP size, structure, and other features on the development of drug resistance in the presence of antibiotics (16, 17). In this study, we investigate the impact of different MP characteristics, including MP concentration, surface composition (between plastics and other materials), size, and surface area, on the *in vitro* development of resistance to ampicillin, ciprofloxacin, doxycycline, and streptomycin in *Escherichia coli*. These antibiotics are broad-spectrum, represent different antibiotic classes, and are readily found in wastewater systems (18). More specifically, we probed if MP concentration (no. plastics/µL), size, surface area, and composition (between plastics types) had any impact on (i) bacterial growth alone, (ii) antibiotic-specific AMR, and (iii) multidrug resistance (MDR) between the four antibiotics. We then examined potential mechanisms behind differences in AMR development, specifically studying biofilm formation. Our results identify that MPs play an important and significant role in the development of AMR as they act as novel surfaces for biofilm-associated resistance. Overall, our findings may provide context to wastewater surveillance data and provide insights into waste management and associated disease burdens.

## MATERIALS AND METHODS

### Strains and culture conditions

*E. coli* MG1655 (ATCC 700926) was used in all experiments. All liquid cultures were grown in lysogeny broth (Miller, LB) medium under shaking conditions at 180 rpm at 37°C. First, MPs (Table S1) were added to the media for 48 h to allow biofilm surface attachment and full maturity (19). Following the initial MP exposure, additional amendments were added, which included subinhibitory levels of ampicillin (Sigma-Aldrich), ciprofloxacin (MP Biomedicals, 86393-32-0), doxycycline (Sigma-Aldrich), and streptomycin (Sigma-Aldrich), as indicated below.

### Growth curve data

**Fig 1:**
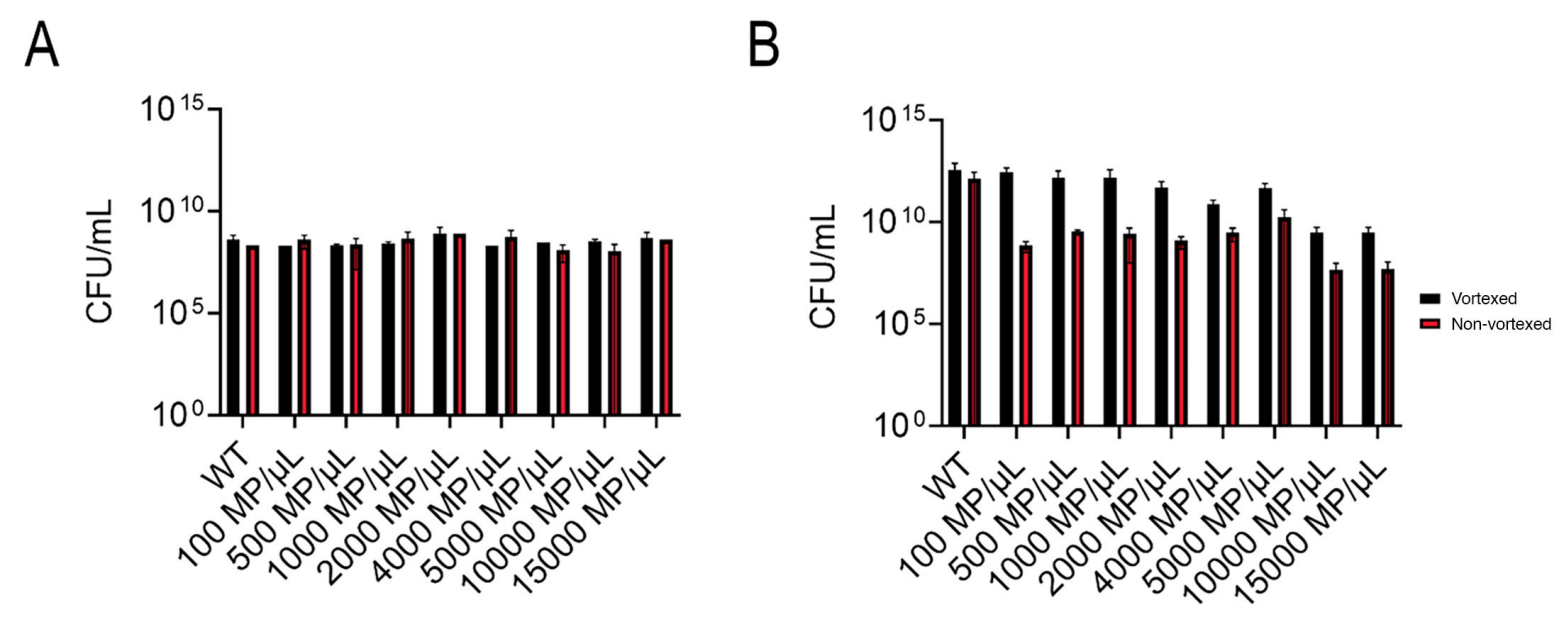
CFU/mL readings after 1 h of growth (**A**) and 24 h of growth (**B**) in WT and MP concentrations (100, 500, 1,000, 2,000, 4,000, 5,000, 10,000, and 15,000 MP/μL; 10 µm diameter PS spheres). Samples that were vortexed are presented in black and non-vortexed samples are depicted in red.

Wild-type (WT) *E. coli* MG1655 was cultured at 37°C in 4 mL of the LB with varying MP concentrations (100, 500, 1,000, 2,000, 4,000, 5,000, 10,000, and 15,000 plastics/μL) in glass culture tubes and sampled at 0, 1, and 24 h. These samples were plated in triplicate on LB agar to determine the CFU/mL at the time point of interest. Cultures were plated before and after being vortexed, to compensate for cells attached to the MP surface, and CFUs were counted 24 h after incubation (Fig. 1)

### Antibiotic and MP susceptibility testing

To determine the initial baseline minimum inhibitory concentration (MIC) of bacteria, cells were grown in drug-free media under shaking conditions with microparticles (MPs and glass) until substantial biofilm growth was detected (~48 h) using confocal microscopy (procedure below). A control sample of cells (without MPs) was also grown for 48 hand their MICs were tested along with the samples exposed to MPs. Microparticles were vortexed for 1 min and spun down to release the biofilm on the surface (Fig. S1). This resulted in both biofilm and planktonic cells in the same culture. The vortexed solution was used in a broth microdilution MIC in a 96-well plate using LB media, effectively testing the MIC of attached cells and effluent cells (Fig. S2) (20). Briefly, for the microbroth dilutions, 100 µL of media with the antibiotic is diluted twofold into the next column and so on. A portion (10 µL) of cells from the sample of interest is then placed in each well and grown in a static incubator for 24 h at 37°C. Following this, the optical density is checked in a spectrophotometer at 600 nm and recorded against a negative control (LB). The MIC was measured every 24 h during the passaging experiment as shown in Fig. S2.

#### MP-antibiotic passaging assays

To test the impact of prolonged exposure to MPs on resistance, we passaged cells with media containing either MPs only, antibiotics only, or MPs and antibiotics together. A control passage in media only was also performed. For the control and antibiotic only samples, after an initial 48-h growth period as described above, saturated liquid cultures (cultures that had reached carrying capacity) were passaged once a day into fresh amended media which contained either the antibiotics only or no antibiotic). This marked day 0 of the passaging experiment. Passaging was done at a 1:100 dilution in 4 mL of LB broth, and antibiotics were added at 40% of the initial MIC measured on day 0 (the beginning of subinhibitory antibiotic exposure, Fig. S2) of the corresponding antibiotic, and their respective growth conditions. The initial MIC values reported represent the day 0 MICs measured for cells at the start of the experiment, and these were used as the baseline for calculating fold changes. For experiments grown with MPs, the day 0 MIC calculation *included* biofilm cells due to the timeframe we allowed them to grow in. While most replicates started with the same MIC, occasional variations occurred, and the 40% MIC used for passaging reflected the starting MIC of each replicate.

For the MP samples (with or without antibiotic), after each 24-h exposure period, samples were vortexed to release a portion of the biofilm cells into the supernatant (Fig. S1). Then, the MPs were spun down (if they were 10 µm) or allowed enough time to settle (between 5 and 10 min), after which the supernatant was removed from the culture tube (with 40 µL saved to passage into the new media and use for the MIC assays), and 4 mL new media (along with 40 µL of the supernatant) was allotted into the *same* culture tube containing the *same* MPs (containing biofilm that remains after vortexing) throughout the entire experiment (Fig. S2). All groups were tested for antibiotic susceptibility every 2 days, as described above. A schematic of the experiment is displayed in Fig. S2. This ultimately passaged biofilm and planktonic cells into the same MPs throughout the experiment.

#### Biofilm and planktonic cell-specific susceptibility tests

To determine the change in MIC of the only planktonic or only biofilm cells (cells attached to the surface of the MP), we altered the serial passaging experiments described above.

Similar to above, WT cells were grown with 40 MP/mL (500 µm diameter PS spheres) for 48 h in shaking conditions at 37°C. The supernatant of unattached cells was then removed, and the MIC of these cells was used as the baseline MIC for the “planktonic cells.” Fresh cell-free media were added to the remaining MPs, and the culture was vortexed. The supernatant, now containing released biofilm cells, was removed. The MIC of these cells was used as the baseline MIC for the “biofilm cells.”

For planktonic cells, the supernatant (before vortexing) from the day 0 samples was passaged into fresh media with fresh MPs for “day 1.” The next day, after the MPs had settled, “day 1” supernatant was passaged into fresh media and fresh MPs for “day 2,” and so on. For biofilm cells, the supernatant was removed each day, and the MPs were resuspended in fresh media and vortexed to release the biofilm cells used in the MIC assay. The vortexed sample was not added back into the experiment.

For testing the MIC of the cells during the experiment, the tested planktonic cells were simply the supernatant of that particular day. The biofilm cells were isolated by taking out the supernatant and resuspending the MPs in 1 mL of fresh media. The sample was then vortexed to release the biofilm cells in the fresh media. A portion (350 µL) of each cell type was taken from its respective culture, and the OD was measured. Due to the concentrated nature of the planktonic cells versus the biofilm cells, the planktonic culture was diluted to match the OD of the biofilm cells. The cells were then tested in the MIC assays described below.

### Confocal laser scanning microscopy (CLSM) image collection and analysis

Visualizing the cells on the surface of the particles was performed using confocal microscopy. Using the cultures described in the susceptibility testing section, 500 µm diameter PS spheres was randomly selected out of each culture and dyed using the LIVE/DEAD *Bac*Light Bacterial Viability Kit Protocol, per the manufacturer’s instructions. Briefly, SYTO 9 was used to analyze viable cells, as it can permeate all bacterial cell membranes. In contrast, propidium iodide was used to count dead cells, as it can only enter cells with disrupted membranes. Each plastic was put into a centrifuge tube with 1 mL of sterile water, and 3 μL of staining solution (1.5 µL of each dye) was injected into the tube. Samples were incubated for 20 min at room temperature and protected from light.

Stained samples were imaged with an inverted CLSM (Olympus DSU spinning disk confocal) using a 10× oil immersion objective. Samples individually stained with propidium iodide and SYTO 9 were first analyzed separately to ensure clear signals without overlap. The particles were removed from the dye and suspended in a costar clear round bottom 96-well plate to preserve the MP shape while imaging. After finding the sample and adjusting brightness parameters, z-stacks with an optimized step size were taken for each sample to obtain a 3D visualization of biofilm viability, starting at the base of the spherical particle where growth started and finishing at the top of the sphere where the growth ended.

Z-stacks were analyzed using FIJI (21). 3D renderings were made by taking the .oir file raw data from the microscope and merging the red and green channels. Following the merge, the channels were elucidated by merging the stacks via image -> stacks -> z project with a “Max Intensity” projection type.

### Biofilm formation and crystal violet staining

*E. coli* was inoculated in a 3–5 mL culture and grown to stationary phase for 24 h. The following day, sterile, nontreated 24-well plates (Celltreat #229524, Celltreat Scientific Products, Pepperell, MA, USA) were inoculated in 400 µL LB medium per well. The plates were covered and incubated statically at 37 °C for 48 h. After the inoculation period, the cells were stained per Merritt et al.’s protocol (20). Well optical densities (ODs) were then measured in a spectrophotometer at a wavelength of 500–600 nm.

### Motility assay

Following the protocol by Partridge and Harshey (22)*,* 0.3% Agar plates were made and poured to the same thickness (20 mL), and let dry for 1 h (23). Using a P20 tip, 5 µL of culture was pipetted, inserted into the agar about halfway through, and dispensed. Plates were incubated at 30°C and the diameter of the radial movement (indicated by the visible growth of cells) was then measured using a ruler in centimeters (cm) after 20 h.

### Statistical analysis

The significance of MIC fold changes was determined using an ordinary one-way analysis of variance (ANOVA). Before performing the ANOVA, the residuals were tested for normality using the Shapiro-Wilk test, confirming a Gaussian distribution. Each variable was then compared to the mean of a control, which varied depending on the study. Multiple comparisons were corrected with the Dunnett test, with *P* values adjusted accordingly. Finally, the residuals were tested for homogeneity of variances and potential clustering through the Brown-Forsythe and Bartlett’s tests. The one-way ANOVA was chosen because it allows for comparing mean MIC fold changes across groups under the assumption of normally distributed residuals, which was confirmed here with the Shapiro-Wilk test. Dunnett’s test was applied for *post hoc* comparisons, as it is well-suited for comparing each group to a control with minimized type I error. Finally, the Brown-Forsythe and Bartlett’s tests were used to assess variance homogeneity, ensuring the robustness of the ANOVA results.

## RESULTS

### MPs impact growth at high concentrations

Eight different concentrations of MPs were grown with *E. coli* for 18 h to determine whether MPs influence bacterial growth. The MPs used for testing concentration dependence were 10 µm diameter PS spheres at concentrations of 100 MP/µL, 500 MP/µL, 1,000 MP/µL, 2,000 MP/µL, 4,000 MP/µL, 5,000 MP/µL, 10,000 MP/µL, and 15,000 MP/µL. Colony-forming units per mL (CFU/mL) were tested at 1 h and 24 h after growth in the different concentrations. After 1 h of growth, there were no significant differences in growth between the concentrations; however, at 24 h, two MP solutions (10,000 and 15,000 MP/μL) showed a significantly lower number of CFU/mL compared to the WT (Fig. 1A and B). Notably, counts were higher in vortexed samples at 24 h, indicating the presence of surface-attached cells on the MPs, which is corroborated by confocal imaging (Fig. S1).

### Exposure to PS MPs increases MDR

**Fig 2:**
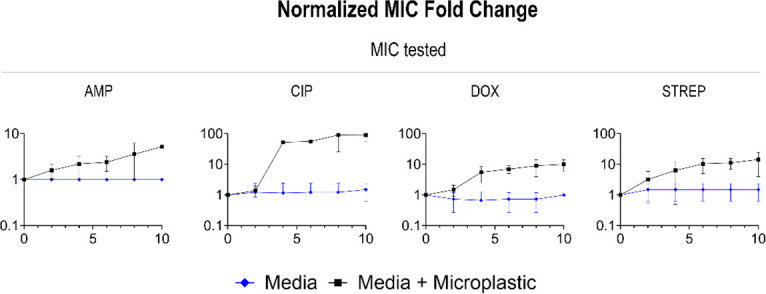
Normalized 10-day time (*x*-axis) series fold change (*y*-axis) of 500 µm diameter PS MPs at 40 MP/mL, grown up in LB and then tested for the MIC in all four antibiotics (left to right: ampicillin, ciprofloxacin, doxycycline, and streptomycin).

We next sought to determine if cells exposed to MPs and subinhibitory levels of a given antibiotic, another common environmental contaminant, had altered patterns of resistance to other classes of antibiotics, rendering the bacteria multidrug-resistant. The four antibiotics we tested were ampicillin (β-lactam antibiotic), ciprofloxacin (fluoroquinolone), doxycycline (tetracycline), and streptomycin (aminoglycoside), which are all commonly found within the environment (18, 24). First, we measured changes in MIC in cells grown with MPs (500 µm diameter PS spheres due to their ease of passaging) alone, to determine the role of MP on AMR in the absence of antibiotics (Fig. 2). In these susceptibility assays, specifically, we are testing the MIC of effluent containing attached cells released from the MPs upon vortexing (Fig. S1).

Strikingly, the presence of MPs alone led to increased resistance to all the antibiotics listed above compared to media containing no MPs. While the MIC of bacteria grown in only LB stayed relatively stable, the samples co-cultured with MPs displayed a significantly higher resistance at a faster rate (Fig. 2). Most notably, the cells from the passaged MPs samples had an almost 100-fold higher MIC by day 10 compared to day 0 for ciprofloxacin, doxycycline, and streptomycin (Table S1). Next, we measured the MIC of cells grown with a single subinhibitory antibiotic with or without MPs (500 µm diameter PS spheres).

**Fig 3:**
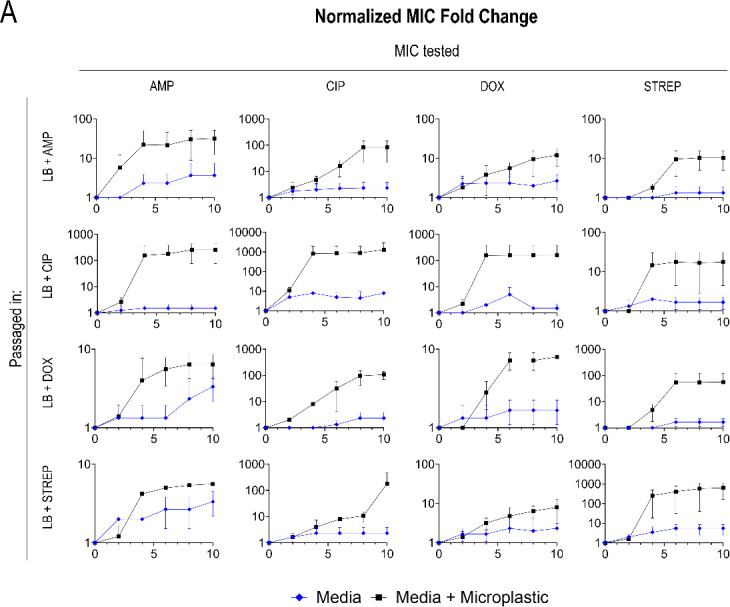
Normalized 10-day time (*x*-axis) series fold change (*y*-axis) of 500 µm diameter PS MPs at 40 MP/mL, grown up in one of four antibiotics left to right: ampicillin, ciprofloxacin, doxycycline, and streptomycin and then tested for the MIC in top to bottom: ampicillin, ciprofloxacin, doxycycline, and streptomycin.

Our results suggest that the addition of MPs led to an increase in AMR for nearly all antibiotics. In the first four rows of Fig. 3, we show the fold change of antibiotics relative to WT for bacteria grown in either antibiotics alone or antibiotics and MPs. In each case where bacteria were grown and tested in the same antibiotic, the addition of MPs to antibiotics in the media led to an MIC increase of at least five times more compared to cells grown in the antibiotics alone. Interestingly, bacteria grown in ciprofloxacin with MPs generally had higher levels of MDR (up to 171 times that of the control grown in the antibiotic) (Fig. 3; Table S1). The other antibiotics with MPs displayed resistance to ciprofloxacin of up to 75-fold higher than the antibiotic control (Table S1). Additionally, bacteria grown in streptomycin with MPs developed extremely high levels of resistance to ciprofloxacin, doxycycline, and streptomycin (Table S1).

After 10 days of exposure to subinhibitory antibiotics, exposure to subinhibitory antibiotics was halted, and bacteria were grown in antibiotic-free media for 5 days. The MIC was tested daily to determine if resistance was stable. Of the 16 conditions, 81.25% (13 out of 16) of the bacteria grown in MPs and subinhibitory antibiotics retained the day 10 resistance to their respective antibiotic or even gained resistance (Fig. S3). Conversely, 43.75% (7 out of 16) of the bacteria grown in just MPs retained or grew in resistance to their respective antibiotics. Surprisingly, only 18.75% (3 out of 16) of the bacteria grown in the subinhibitory antibiotics alone retained resistance. Two of the three conditions that expressed stable resistance were grown in doxycycline (Fig. S3).

### Exposure to different plastic characteristics (concentration and size) does not affect resistance

Next, we sought to investigate whether different MP characteristics (namely size and concentration) affected antibiotic resistance. For simplicity, we used ciprofloxacin in these experiments as the antibiotic showed the highest resistance changes in the abovementioned studies. Thus, the effect of MP concentration (number of plastics/μL) on the development of ciprofloxacin resistance was investigated under subinhibitory ciprofloxacin conditions. To empirically understand whether the concentration of MPs affected ciprofloxacin resistance development and magnitude of resistance, we exposed the bacteria to subinhibitory levels of ciprofloxacin (7.5e−6 mg/mL, 40% of the initial MIC detailed above) and different concentrations of PS MPs. Resistance was determined in MIC fold changes relative to the control (WT *E. coli* grown without MPs or subinhibitory levels of antibiotics) which were tested every 48 h.

**Fig 4:**
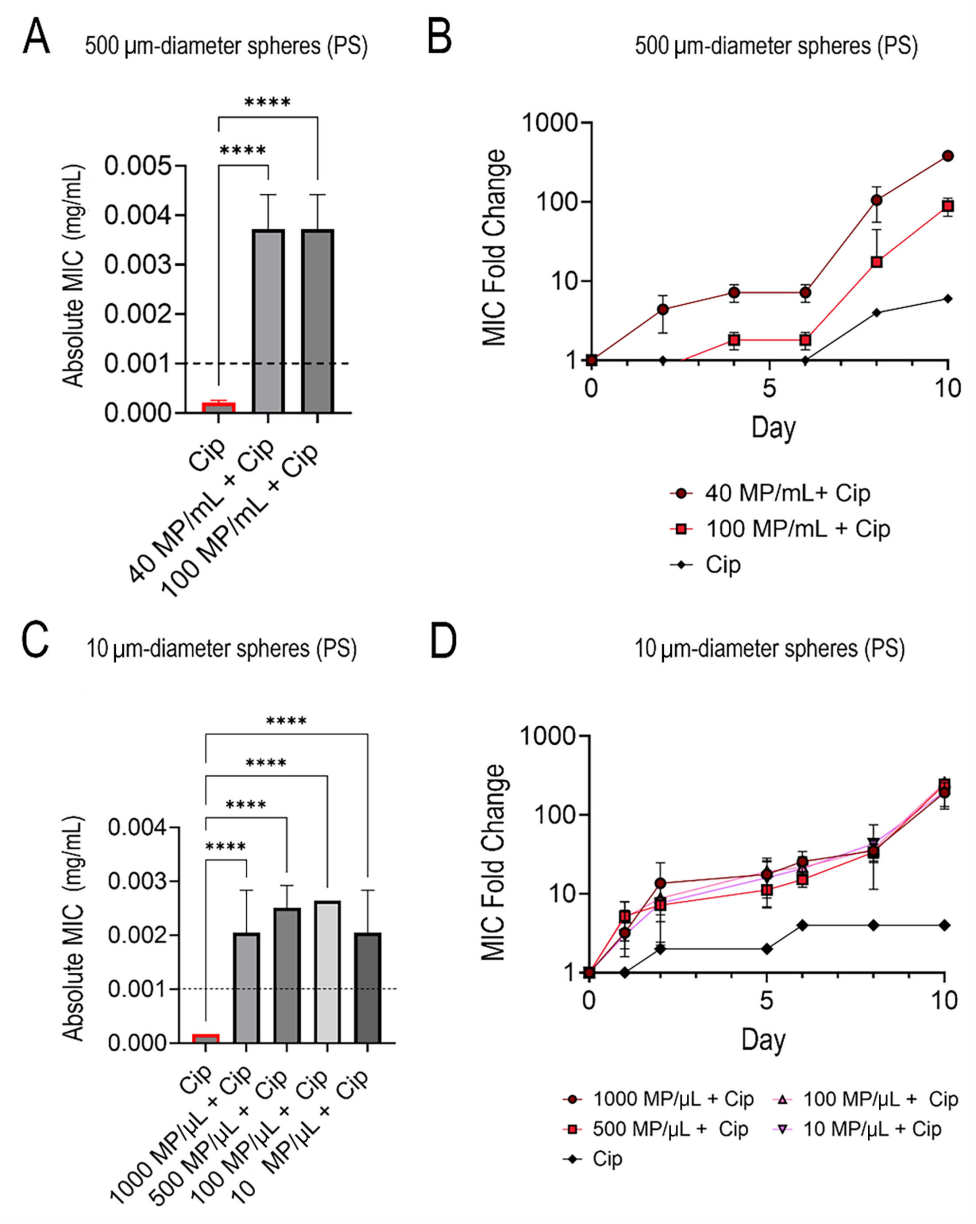
Absolute ciprofloxacin MIC values for 500 µm diameter PS MPs at two different MP concentrations with the dashed line indicating the ciprofloxacin clinical breakpoint concentration at day 10 of exposure (A), time series fold change of 500 µm diameter PS MPs at different MP concentrations and the subinhibitory antibiotic control (ciprofloxacin) relative to the WT (B), absolute ciprofloxacin MIC values for 10 µm diameter PS MPs at four different MP concentrations with the dashed line indicating the ciprofloxacin clinical breakpoint concentration at day 10 of exposure (C), time series fold change of 10 µm diameter PS MPs at different MP concentrations and the subinhibitory antibiotic control (ciprofloxacin) relative to the WT (D).

The concentrations of MPs tested with the 500 µm diameter PS spheres were 40 and 100 MP/mL. Conversely, the concentrations tested with the 10 µm PS diameter beads were 1,000, 500, 100, and 10 MP/μL. There was a significant difference in ciprofloxacin MIC fold changes between each concentration of MP and the MP-free counterpart (bacteria with subinhibitory levels of ciprofloxacin but no MPs) as well as the WT (no MPs or antibiotics introduced during growth) (Fig. 4A and C). However, there was no significant difference in changes in ciprofloxacin MIC between the different concentrations of MPs (Fig. 4B and D); however, the absolute MIC values increased up to 3.713 ± 0.66 µg/mL of ciprofloxacin—over three times the Clinical & Laboratory Standards Institute (CLSI) defined 1 µg/mL clinical breakpoint—for the larger diameter beads and 2.508 ± 4 µg/mL of ciprofloxacin for the smaller beads (Tables S3 and S4) (20).

**Fig 5:**
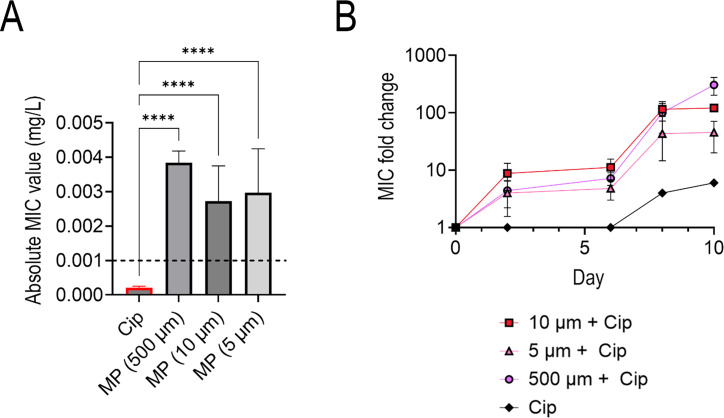
Absolute ciprofloxacin MIC values for various sized PS MPs at 100 MP/μL with the dashed line indicating the ciprofloxacin clinical breakpoint concentration at day 10 of exposure (**A**), time series fold change of various sized PS MPs at 100 MP/μL, and the subinhibitory antibiotic control relative to the WT (**B**).

MIC variation based on MPs’ size and surface area was investigated following the results above. The sizes of PS MPs investigated were 5 µm (100 MP/μL), 10 µm (100 MP/μL), and 500 µm (40 MP/μL) diameter spheres. All three size variations were exposed to subinhibitory ciprofloxacin along with a control that had no MPs added. Results showed that the difference between the MPs themselves had no significant statistical difference. While the results showed no significant difference between the different surface areas of the beads, the cells grown with MPs and antibiotics again consistently had higher absolute MIC values (above the clinical breakpoint) compared to the antibiotic-only comparators (Fig. 5A and B) and the WT.

### Plastic compositions affect antibiotic resistance

**Fig 6:**
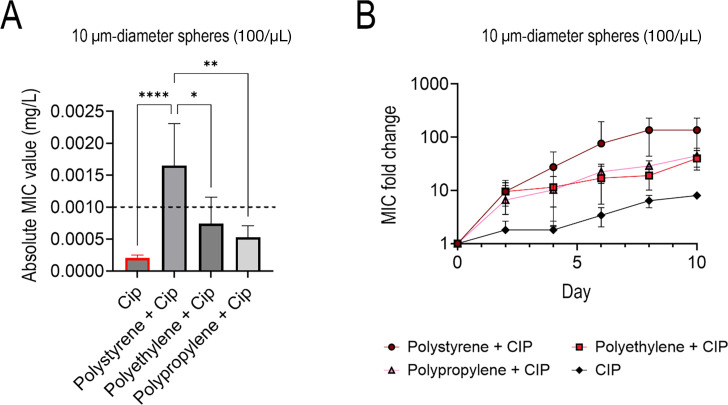
Absolute ciprofloxacin MIC values for 10 µm diameter PS, PE, and PP MPs at 100 MP/μL with the dashed line indicating the ciprofloxacin clinical breakpoint concentration at day 10 of exposure (**A**), time series fold change of 10 µm diameter PS, PE, PP MPs at 100 MP/μL, and the subinhibitory antibiotic control relative to the WT (**B**).

Next, we sought to determine if plastic composition affected the development and magnitude of ciprofloxacin resistance (Fig. 6). This experimental design tested and compared the three most common plastic types—PS, PE, and PP (25). We found that all three conditions with varying MP compositions had a significantly higher MIC after 10 days of exposure than the MP-free control exposed to only subinhibitory levels of ciprofloxacin. The PS samples reached a significantly different absolute MIC value (1.65 ± 0.572 µg/mL) than PE (0.743 ± 0.36 µg/mL), PP (0.528 ± 0.162 µg/mL), and the control of only ciprofloxacin (0.20625 ± 0.04 µg/mL) (Fig. 6; Table S5). Furthermore, the PS MPs facilitated an absolute MIC higher than the ciprofloxacin-*E. coli* clinical breakpoint (1 µg/mL). Interestingly, the rates of AMR growth in all three plastic samples were similar in terms of fold change (Fig. 6B).

### Increased resistance and biofilm on PS compared to glass

**Fig 7:**
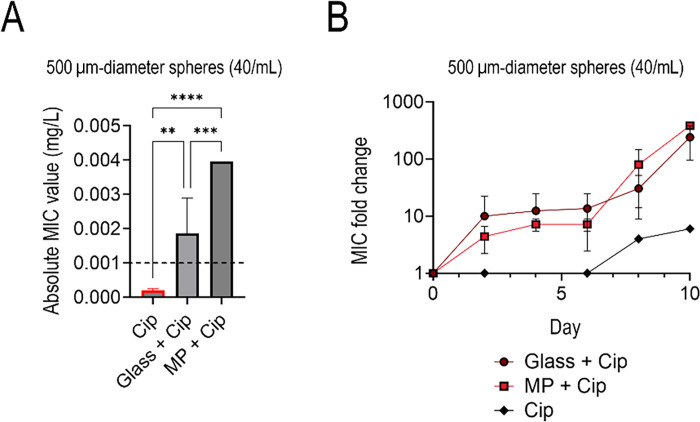
Absolute ciprofloxacin MIC values for 500 µm diameter PS and glass spheres at 40 MP/mL at day 10 of exposure (**A**), with the dashed line indicating the ciprofloxacin clinical breakpoint concentration. Time series fold change of the 500 µm (**B**) glass, PS, and the subinhibitory antibiotic control relative to the WT.

We also studied the difference in resistance development between PS MP and glass spheres (500 µm diameter spheres at the same concentration of 40 MP/μL) to determine if plastic substrates had a specific effect or if any small particles would lead to an increase in AMR (Fig. 7). Similar to previous experiments, the microparticles were exposed to subinhibitory levels of ciprofloxacin and compared to a control, which only had the subinhibitory ciprofloxacin and no glass or plastic particles. The PS plastic spheres facilitated a statistically higher absolute MIC value by the end of the 10-day study from its glass counterpart and antibiotic control (Fig. 7A). Like Fig. 6, the fold changes of the attachment surfaces were not statistically different (Fig. 7B). This indicates that the rate of AMR growth is similar in terms of fold change.

**Fig 8:**
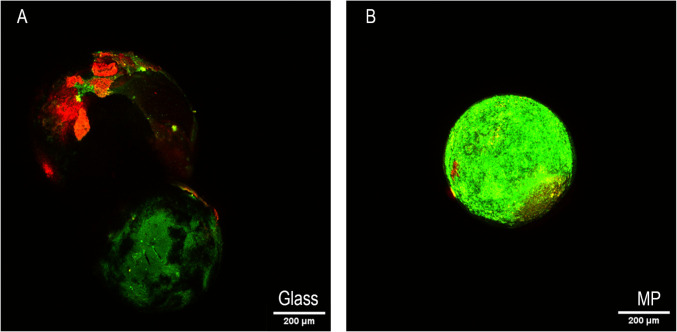
CLSM image of 500 µm diameter glass (A) and PS (B) spheres at day 10 of subinhibitory ciprofloxacin exposure. Green pixels indicate live cells, while red indicates dead cells.

To elucidate the mechanism behind the high levels of resistance found in the samples containing the MPs (particularly PS, Fig. 2 and 3) and the discrepancies behind the resistance profiles of the particles of different compositions, we wanted to visualize the surface of the particles. We did this qualitatively by using confocal microscopy. The glass and PS spheres were dyed on day 10 of subinhibitory ciprofloxacin exposure and compared to each other (Fig. 8) using a live dead stain. Green pixels indicate viable growth (e.g., live cells). In contrast, red indicates nonviable growth (dead cells). The glass (Fig. 8A) depicts two spheres, each having a significantly decreased amount of growth on the surface of the sphere, and most of the growth exhibited was colonized by dead cells. Conversely, the PS condition had mostly live cells, and bacteria colonized the whole surface.

### Exposure to MPs selects for increased biofilm

**Fig 9:**
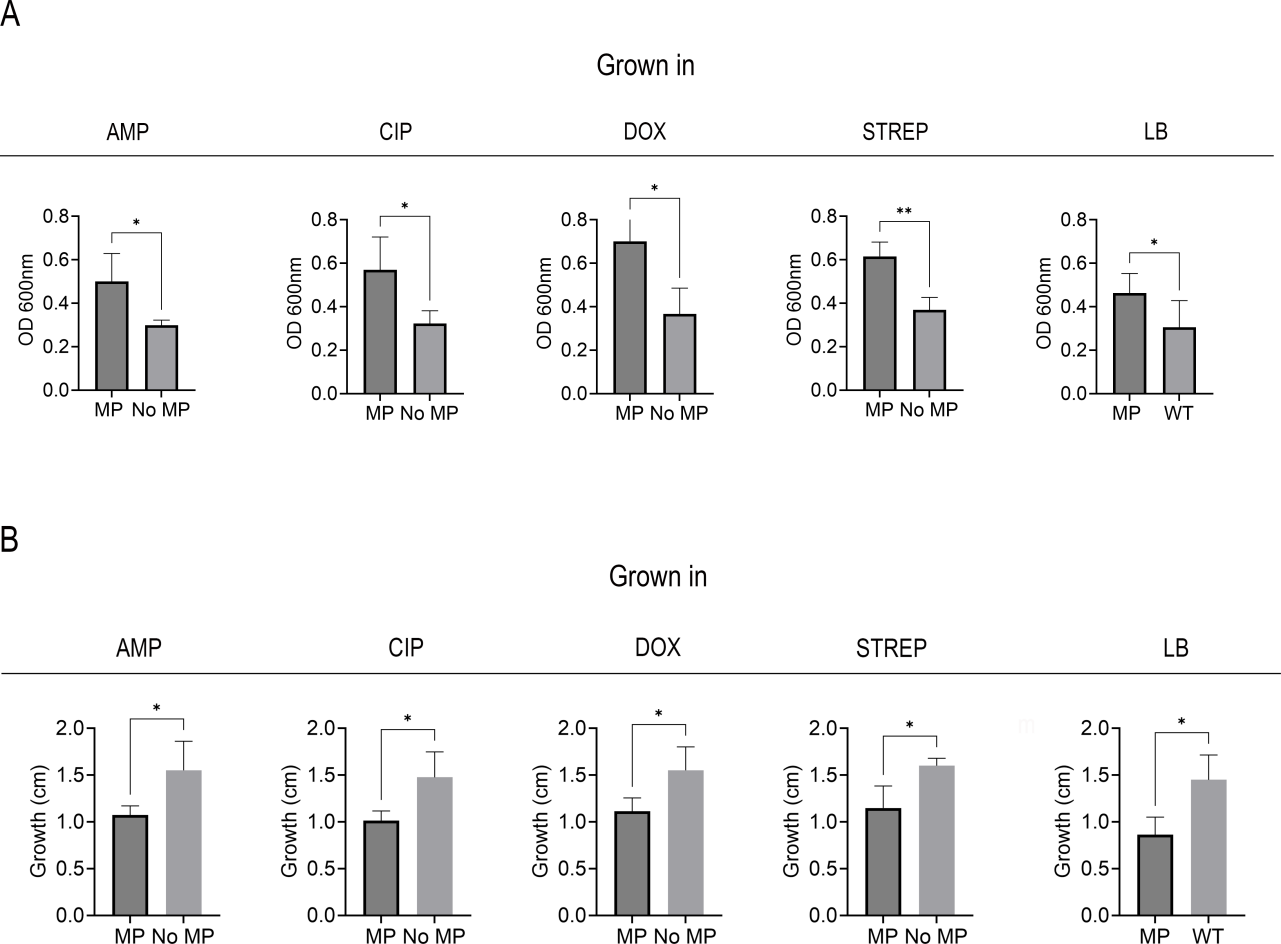
OD readings of crystal violet staining for *E. coli* grown with or without MPs in media containing subinhibitory levels of four antibiotics left to right: ampicillin, ciprofloxacin, doxycycline, or streptomycin or no antibiotics (LB) (**A**). Motility in centimeters (cm) of *E. coli* grown with or without MPs in media containing subinhibitory levels of four antibiotics left to right: ampicillin, ciprofloxacin, doxycycline, or streptomycin or no antibiotics (LB) (**B**).

Following the visualization of robust biofilms on the surfaces of the PS MPs, we sought to understand the role of biofilm further. Specifically, we investigated whether cells passaged with MPs (Fig. 2 and 3) formed more robust biofilms in the absence of MPs (i.e., does the presence of MPs select for better biofilm formers). Crystal violet staining can detect biofilm formation as it binds to bacterial cells and the extracellular matrix, making biofilms both visible and quantifiable. The bacteria grown with and without MPs along with various antibiotics and controls for 10 days (Fig. 2 and 3) were purified of MPs and trace antibiotics and grown in a 24-well PS dish for 48 h, and biofilm (surface attachment) was quantified using 0.1% crystal violet solution (Fig. 9A). Bacterial motility is a broad mechanism involved in biofilm formation, specifically, impaired motility is associated with increased biofilm due to several interconnected factors, mainly related to regulatory shifts, changes in surface properties, and environmental sensing mechanisms (26). Thus, swimming motility was also assayed using the bacteria grown with and without MPs along with various antibiotics and controls for 10 days (Fig. 2 and 3) on 0.3% soft agar (Fig. 9B).

**Fig 10:**
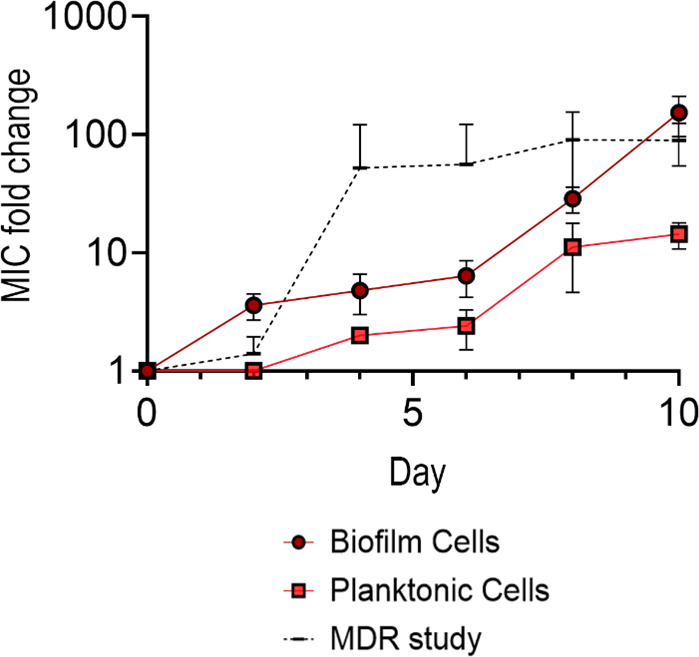
Time series ciprofloxacin fold change of *E. coli* biofilm and planktonic cells exposed to 500 µm PS MP relative to WT, and the MDR study of *E. coli* grown with MP/LB combination tested in ciprofloxacin (dashed line).

All samples grown with MPs had significantly more biofilm growth than those grown without MPs, indicated by their higher OD readings and increase in stained surface-attached cells (Fig. 9A; Fig. S4). The cells not grown with MPs had significantly larger diameters than those grown with MPs, indicating that the bacteria exposed to MPs had impaired motility (Fig. 9B; Fig. S5). As our experimental samples tested the MIC of a combination of surface-attached (biofilm) and planktonic cells, we wanted to assess the specific contributions of biofilm and planktonic cells to the observed increase in AMR. We thus specifically measured the MIC of MP biofilm cells and planktonic cells continuously exposed to MPs over 10 days. Biofilm MICs tested in ciprofloxacin showed increases in MIC like those of cells grown in LB with MPs from the MDR study (Fig. 10) (*P* = 0.1175) with day 10 values averaging at 153.6-fold change from day 1 MIC values (Fig. 9; Table S7). The planktonic cells exposed to MPs and WT cells (no MPs) both differed significantly from the MDR study values (*P* < 0.001 and <0.001, respectively), but not from each other. These data suggest that the cells from the biofilm are the primary contributor to the observed increase in observed prior experiments (Fig. 10).

MPs are significant environmental pollutants with critical implications for public health, particularly in the context of increasing AMR. Understanding how MPs affect the development of AMR is a crucial step in better understanding how environmental factors shape AMR in bacteria. This will only become more important as both MPs and AMR become more prevalent. Our study showed that the presence of MPs directly correlated with an increase in biofilm-associated AMR rate of development and its magnitude. More specifically, MPs were associated with post-breakpoint MDR to four distinct families of antibiotics (Fig. 2A and B). Breakpoints are an integral part of modern microbiology practice and define susceptibility and resistance to antibacterials in the clinical setting (e.g., related to human health) (27).

PS, in particular, was associated with the most significant impact on resistance development, which was surprising given its relative hydrophilicity compared to PE and PP (Fig. 6). Previous studies have shown that bacteria have an affinity for plastic due to their high hydrophobicity and oxidation, which can lead to easy adhesion (28, 29). However, it is essential to note that studies have shown that *E. coli* prefers hydrophilic surfaces over hydrophobic ones (30). Of the three plastics used in this study, PS is the most hydrophilic, while PP is the most hydrophobic, so these findings are in line with *E. coli’s* preference to hydrophilic surfaces. With this in mind, we expected glass beads of similar diameter to have a higher adhesion rate and, therefore, a higher resistance to the tested antimicrobials. This, however, was not the case, as PS had a higher absolute MIC value and greater MIC fold change over the glass condition. This indicates that plastics may be a unique substrate for bacteria to develop and maintain resistance to.

While the complete mechanism is not yet known for AMR on MPs, the current prevailing theory indicates that biofilm formation upon the plastics allows for higher resistance rates (11). As depicted in the CLSM images in Fig. 8, we observed a dense biofilm that spans the entire surface of the MPs, compared to the glass substrate, which has uneven clusters of cells on the surface, with a large portion of them nonviable post-subinhibitory antibiotic exposure. This would help explain the differences in MIC shown in Fig. 7. We investigated the effects the cells had post-MP exposure to see if they had a propensity for forming biofilms over their counterparts grown in the same media without MPs. Qualitatively, confocal imaging found that not only did the bacteria grown with MPs develop biofilms, but they also grew biofilms with more biomass on the plastics than on glass surfaces (Fig. 8). Notably, cells grown in subinhibitory levels of doxycycline and MPs had the highest biofilm growth. Doxycycline was the only bacteriostatic antibiotic—an antibiotic that attacks cell reproduction rather than the cell itself—used in this study. Doxycycline is known to target the 30S ribosomal subunit and inhibit protein synthesis, which can trigger stress pathways that upregulate biofilm-associated genes and extra polymeric substance production (19). We next examined bacterial motility to investigate further the bacteria’s ability to grow biofilms. In these experiments, we found that samples grown with MPs had impaired motility, which can influence biofilm formation (Fig. 9B) (26). Overall, these data suggest that the presence of MPs select for better biofilm formers, and these cells display increased resistance. These findings are corroborated by Fig. 10, which illustrates that biofilm cells are the primary contributors to the observed increase in AMR in prior experiments. Specifically, biofilm MICs for ciprofloxacin increased significantly over 10 days, mirroring trends from the MDR study, whereas planktonic cells exposed to MPs did not exhibit similar increases, underscoring the dominant role of biofilms in driving resistance. We hypothesize that, especially in the presence of low levels of antibiotics, there is a combinatorial effect between selection for AMR target genes and biofilm formation that leads to high levels of resistance and recalcitrance. Further research is needed to investigate the cellular-level interactions and material properties of plastics that contribute to such high resistance rates.

It is well known that biofilms play a crucial role in the spread of AMR. Bacteria within biofilms produce persister cells that are metabolically inert, which is one mechanism for evading antibiotics (31). These cells can survive even in high concentrations of antibiotics (31). Furthermore, current research suggests that biofilms act as refugia of MDR plasmids by retaining them, even in the absence of antibiotics (32). This would support our results with respect to the MP properties we investigated. First, MP concentration was not factored into different resistance rates. Instead, size and composition had more of an impact on the rate of resistance development and magnitude of resistance. A larger-sized MP would have a larger biofilm and, therefore, a greater capacity to develop resistance. The plastic composition can also play a role in both bacterial attachment and biofilm growth. MPs are known to serve as electron donors for bacterial biofilms to feed on, inducing a faster or easier attachment of the bacteria to the surface and promoting bacterial growth and colonization (22, 28). This may explain the higher concentration of biofilm on PS MP compared to glass (Fig. 7), which in turn would explain PS’s larger resistance load.

The proposed mechanisms of biofilms and selective pressures are assumed to have accumulated overtime and compounded off of each other, creating high resistance levels as the time trial went on. We believe that the potential ramifications of high-level multidrug-resistant bacteria facilitated by biofilms on the surface of the MPs are significant. Moreover, we found bacteria exhibiting this behavior within 10 days of subinhibitory antibiotics and MP exposure. The rate of AMR development and the surpassing of clinical breakpoints in both single and multidrug tests highlight a need to monitor MPs, MP-associated biofilms, and antibiotic levels in the environment. This is especially true in areas with inadequate waste disposal and substandard public health infrastructure, such as low-and middle-income countries (LMICs) and vulnerable populations (33). Future studies in this area should focus on the disparities of wastewater treatment in LMICs and high-income countries and how the different environmental factors shape AMR development. Additionally, wastewater and environmental monitoring should also include the presence of MPs, as they have the potential to exacerbate AMR outbreaks. Our work can inform the ongoing development of AMR surveillance strategies, helping to predict and prevent future outbreaks.

## Data Availability

All data used for this study have been included in the article or supplemental material.

## SUPPLEMENTAL MATERIAL

Supplementary Material 1 aem.02282-24-s0001.docx Legends for Fig. S1 to S5; Tables S1 to S7.

Fig S1 aem.02282-24-s0002.tif MP before and after the sample was vortexed for 1 min.

Fig S2 aem.02282-24-s0003.tif Experimental schematic.

Fig S3 aem.02282-24-s0004.tif Five-day resistance stability was measured in fold change (y-axis) relative to day 10 of the MDR study above.

Fig S4 aem.02282-24-s0005.tif 0.1% crystal violet stains on bacterial samples post 10-day exposure to various media and with or without MPs.

Fig S5 aem.02282-24-s0006.tif Sample of soft agar plates to determine motility of the bacterial samples post 10-day exposure to various media and with or without MPs.

ASM does not own the copyrights to Supplemental Material that may be linked to, or accessed through, an article. The authors have granted ASM a non-exclusive, world-wide license to publish the Supplemental Material files. Please contact the corresponding author directly for reuse.
