# Supplementary figures and images for: Effects of microplastic concentration, composition, and size on Escherichia coli biofilm-associated antimicrobial resistance
Source: Appl Environ Microbiol. 2025 Mar 11;91(4):e02282-24. doi: 10.1128/aem.02282-24 (PMC12016508; doi:10.1128/aem.02282-24)

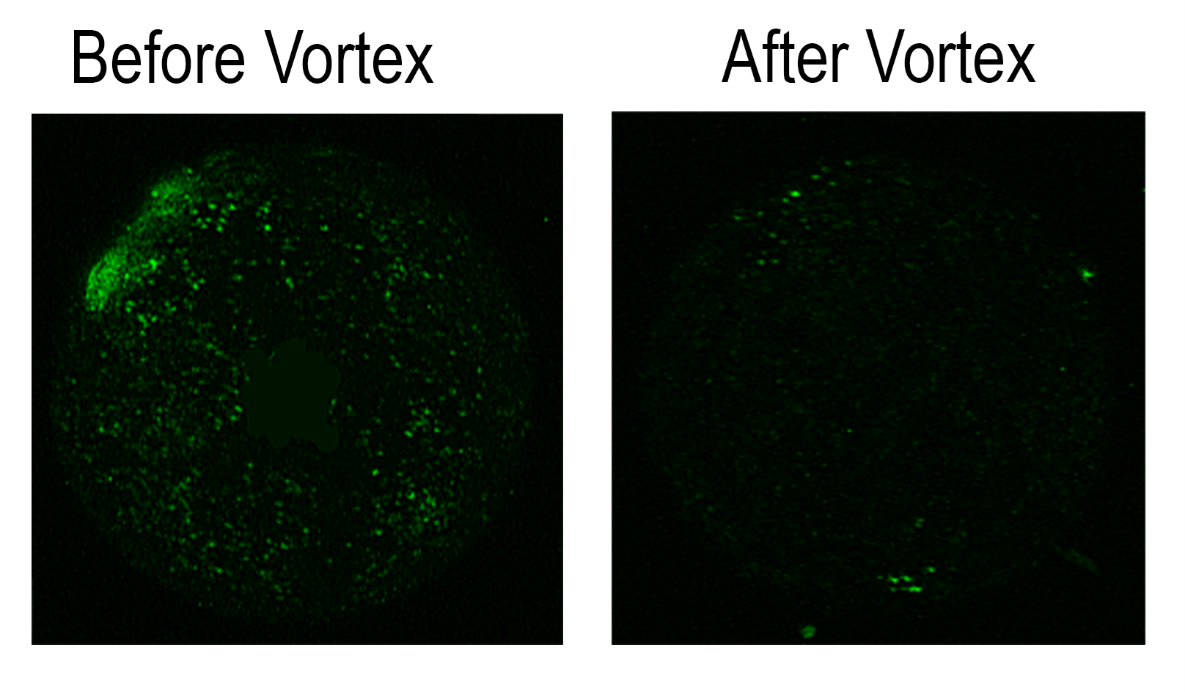

Supplement: Fig S1 — MP before and after the sample was vortexed for 1 min. [file aem.02282-24-s0002.tif]

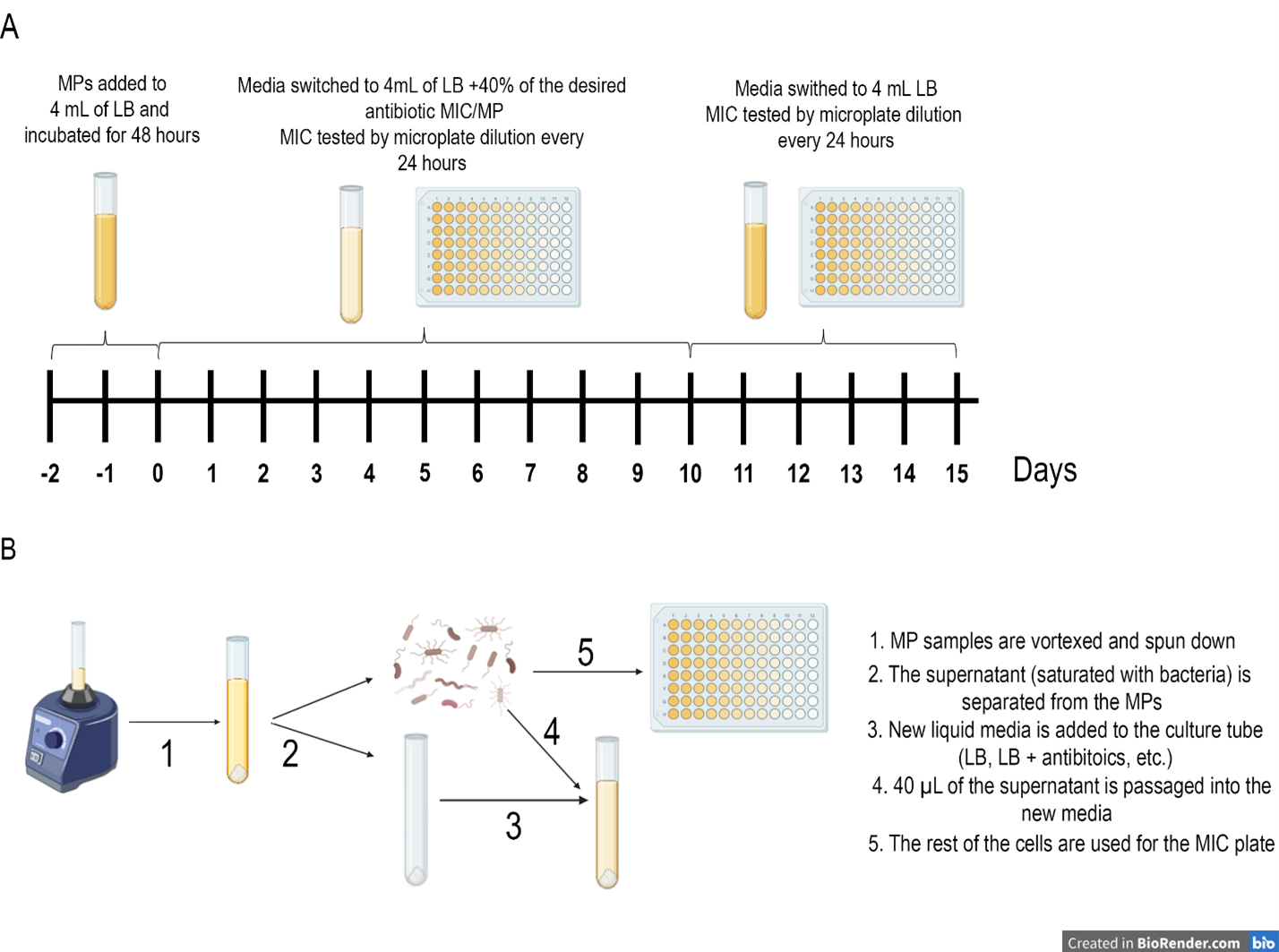

Supplement: Fig S2 — Experimental schematic. [file aem.02282-24-s0003.tif]

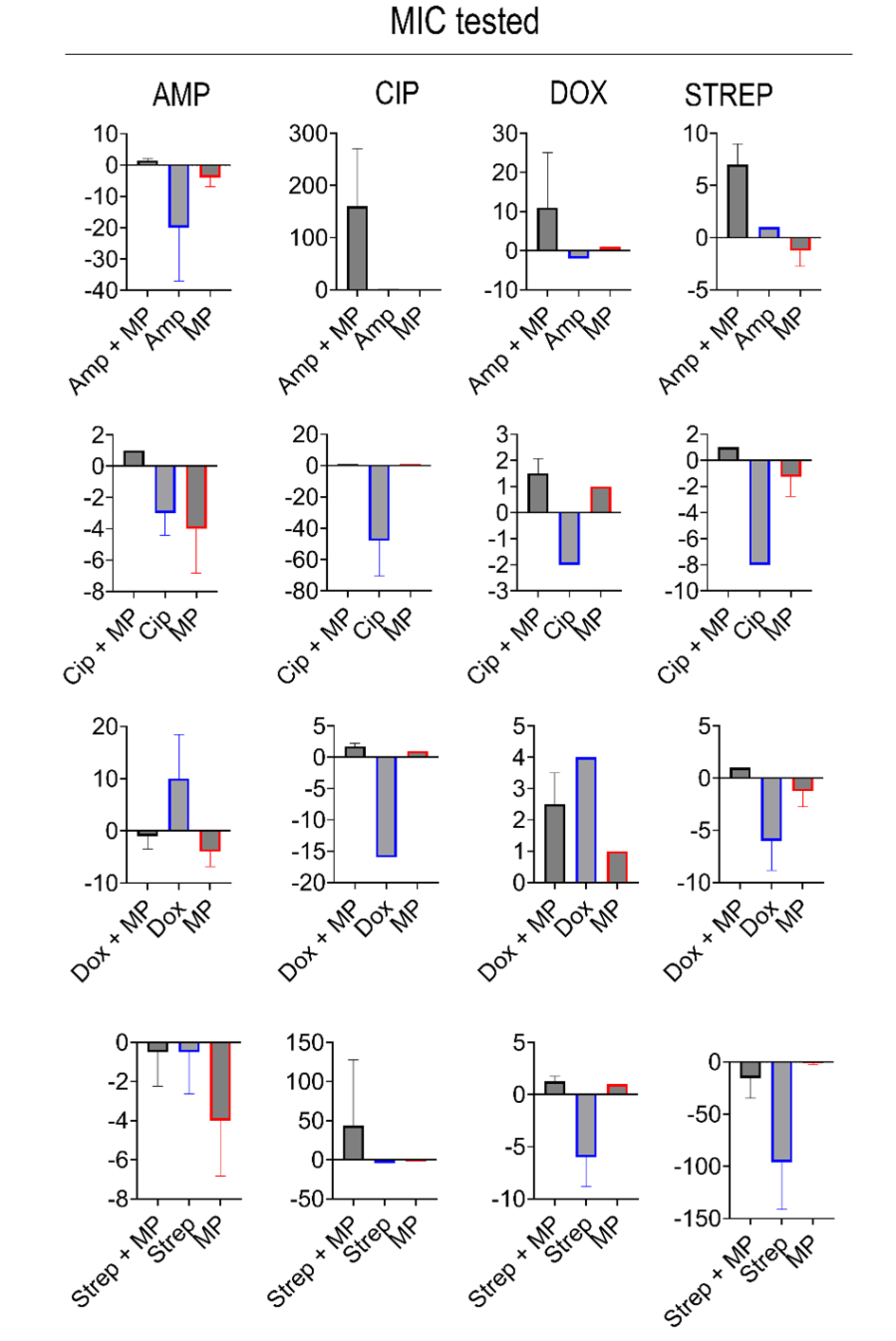

Supplement: Fig S3 — Five-day resistance stability was measured in fold change (y-axis) relative to day 10 of the MDR study above. [file aem.02282-24-s0004.tif]

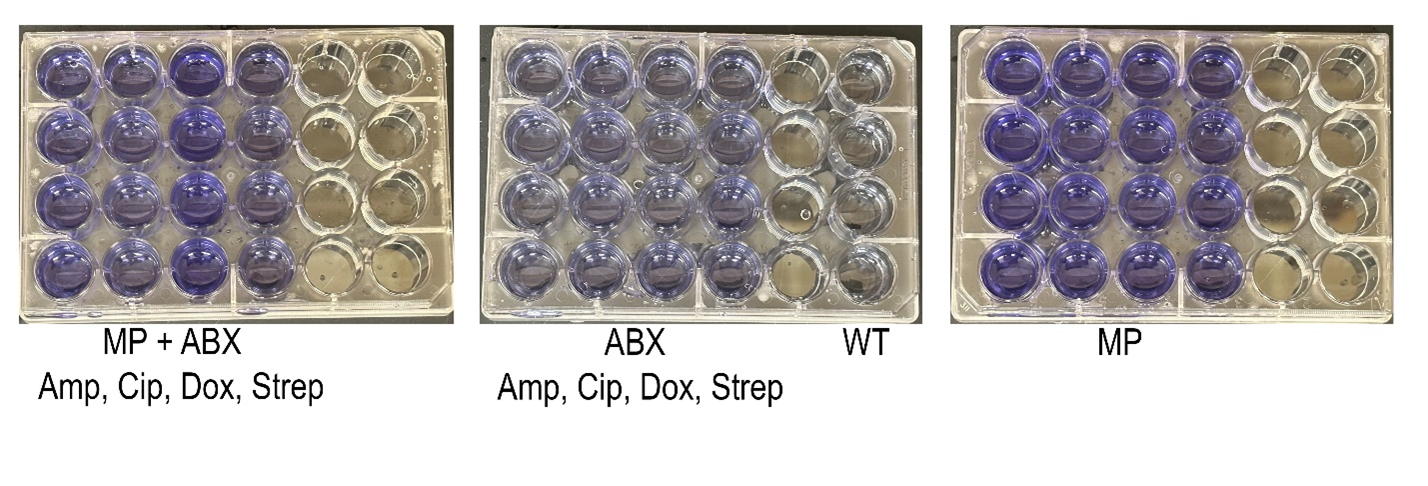

Supplement: Fig S4 — 0.1% crystal violet stains on bacterial samples post 10-day exposure to various media and with or without MPs. [file aem.02282-24-s0005.tif]

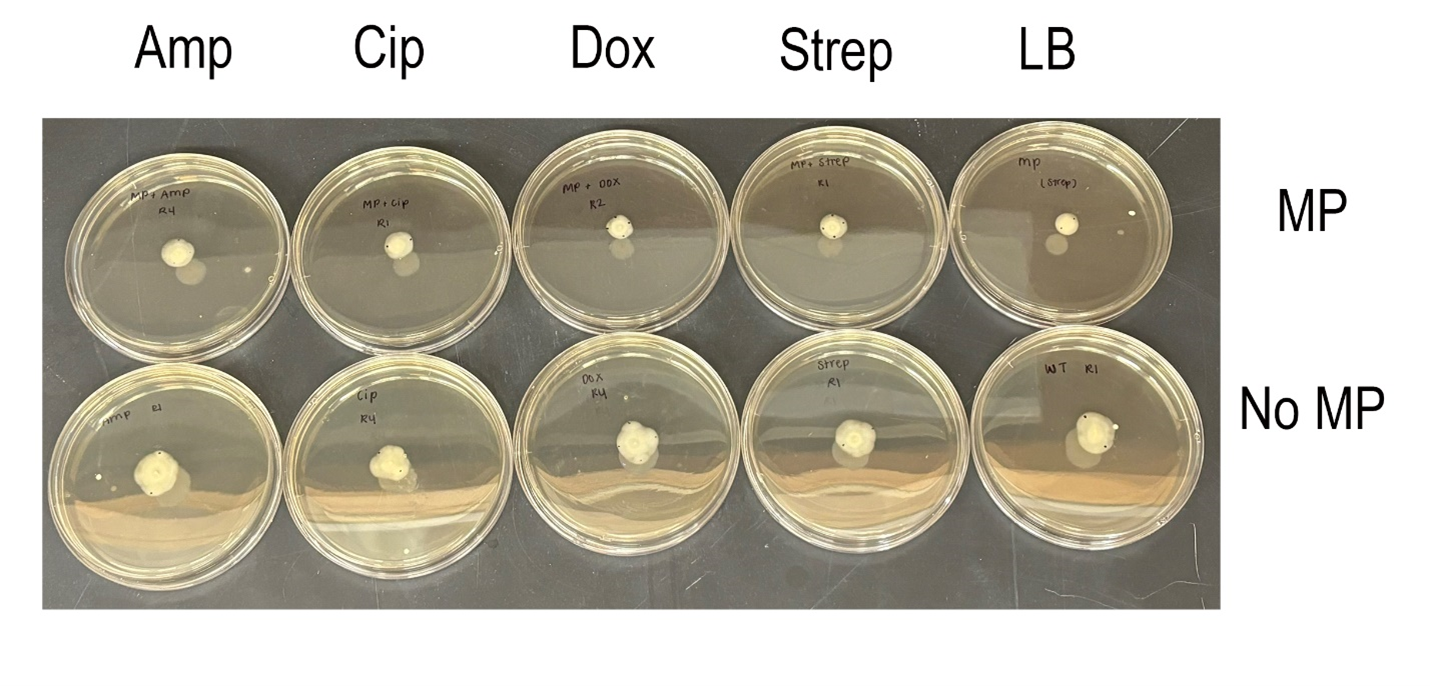

Supplement: Fig S5 — Sample of soft agar plates to determine motility of the bacterial samples post 10-day exposure to various media and with or without MPs. [file aem.02282-24-s0006.tif]
